# Chloroplast Iron Transport Proteins – Function and Impact on Plant Physiology

**DOI:** 10.3389/fpls.2016.00178

**Published:** 2016-02-19

**Authors:** Ana F. López-Millán, Daniela Duy, Katrin Philippar

**Affiliations:** ^1^Department of Pediatrics, Children’s Nutrition Research Center, Baylor College of Medicine, United States Department of Agriculture/Agricultural Research Service, HoustonTX, USA; ^2^Plastid Fatty Acid and Iron Transport – Plant Biochemistry and Physiology, Department Biology I, Ludwig-Maximilians-University of MunichMunich, Germany

**Keywords:** chloroplast, iron transport, metal homeostasis, membrane protein, transporter

## Abstract

Chloroplasts originated about three billion years ago by endosymbiosis of an ancestor of today’s cyanobacteria with a mitochondria-containing host cell. During evolution chloroplasts of higher plants established as the site for photosynthesis and thus became the basis for all life dependent on oxygen and carbohydrate supply. To fulfill this task, plastid organelles are loaded with the transition metals iron, copper, and manganese, which due to their redox properties are essential for photosynthetic electron transport. In consequence, chloroplasts for example represent the iron-richest system in plant cells. However, improvement of oxygenic photosynthesis in turn required adaptation of metal transport and homeostasis since metal-catalyzed generation of reactive oxygen species (ROS) causes oxidative damage. This is most acute in chloroplasts, where radicals and transition metals are side by side and ROS-production is a usual feature of photosynthetic electron transport. Thus, on the one hand when bound by proteins, chloroplast-intrinsic metals are a prerequisite for photoautotrophic life, but on the other hand become toxic when present in their highly reactive, radical generating, free ionic forms. In consequence, transport, storage and cofactor-assembly of metal ions in plastids have to be tightly controlled and are crucial throughout plant growth and development. In the recent years, proteins for iron transport have been isolated from chloroplast envelope membranes. Here, we discuss their putative functions and impact on cellular metal homeostasis as well as photosynthetic performance and plant metabolism. We further consider the potential of proteomic analyses to identify new players in the field.

## Introduction

Chloroplasts, which are unique and highly specialized organelles, originated from the endosymbiosis of an ancestor of today’s cyanobacteria with a mitochondria-containing host cell (Gould et al., 2008). In higher plants, chloroplasts perform essential functions of primary and secondary metabolism, but first and foremost are the site of photosynthesis and thus represent the basis for all life dependent on atmospheric oxygen and carbohydrate supply. In addition to mature, autotrophic chloroplast of green leaves, the plastid organelle family includes many specialist types with manifold biosynthetic functions in certain tissues and developmental stages (e.g., proplastids, etioplasts, elaioplasts, gerontoplasts or chromoplasts). Since, however, due to experimental accessibility most research has been performed on chloroplasts, in the following we will simply refer to plastids, if besides chloroplasts other organelle types are involved. Nevertheless, all metabolic functions of plastids require different selective transport mechanisms across the outer and inner envelope (IE) membranes of the organelle.

In plant cells, the transition metal iron (Fe) plays a major role in redox reactions and as cofactor of many proteins due to its potential for valency changes (Raven et al., 1999). In chloroplasts, Fe is an important component of the photosynthetic apparatus - i.e., found in all electron transfer complexes (PSII, PSI, cytochrome *b_6_f* complex, and ferredoxins) - and is required for biogenesis of cofactors such as heme and Fe–S cluster (for overview see Balk and Schaedler, 2014; Briat et al., 2015). Besides being essential components of the photosynthetic electron transport, Fe-cofactor containing proteins are also involved in protein import and chlorophyll biosynthesis. Chloroplasts represent the Fe-richest organelle in plant cells containing 80–90% of the Fe found in leaf cells (Terry and Low, 1982). However, excess Fe generates ROS, which cause oxidative damage (for overview Briat et al., 2010b). In chloroplasts, this situation is most prominent, since Fe and ROS, like hydrogen peroxide (H_2_O_2_) produced by the photosynthetic electron chain are in close proximity (Asada, 1999; Mubarakshina et al., 2010). In consequence, free unbound Fe leads to the formation of hydroxyl radicals via the Fenton reaction (Halliwell and Gutteridge, 1992). On the other hand, plastid Fe-deficiency impairs chlorophyll biosynthesis, leads to leaf chlorosis and requires remodeling of the photosynthetic apparatus (Spiller and Terry, 1980; Moseley et al., 2002). Chloroplasts suffering from Fe starvation are specifically impaired in proper function of photosystem I (PSI), which contains 12 Fe atoms per monomer. Thus, to maintain cellular Fe-homeostasis and guarantee proper plant development and growth, Fe transport into and out of plastids as well as Fe storage and buffering of free Fe within these organelles need to be tightly controlled (Abadía et al., 2011; Briat et al., 2015). Furthermore, besides in the plastid family, strict control of Fe homeostasis occurs within the plant cell, other organelles and throughout all organs (see Thomine and Vert, 2013).

In this review we will first present an overview of the characteristics of chloroplast Fe-acquisition known so far. Next we describe the characteristics and function of proteins involved in chloroplast Fe transport. Since this transition metal plays an important role not only in many processes in plastid organelles, but also for plant performance in general, we discuss the impact of plastid Fe-homeostasis and transport on plant physiology.

## Chloroplast Iron Acquisition

The mechanisms by which Fe is obtained by chloroplasts are not as well-known as the two Fe-acquisition strategies - reduction-based (strategy I) and chelation based (strategy II) - across the plasma membrane of root cells (for overview see Morrissey and Guerinot, 2009; Kobayashi and Nishizawa, 2012; Brumbarova et al., 2015). Nearly two decades ago, uptake experiments with isolated intact chloroplasts and purified IE membrane vesicles described the existence of a light-dependent plastid uptake of ^59^Fe(III), chelated by epihydrohymugineic acid in barley - a strategy II plant (Bughio et al., 1997). The absorption of Fe by illuminated chloroplasts was inhibited by 3-(3,4-dichlorophenyl)-1,1-dimethylurea, an inhibitor of photosystem II, suggesting that Fe absorption depends upon electron transport or the ATP generated in thylakoids. On the other hand, in the strategy I plant pea, an inward Fe^2+^ transport accross the IE of the chloroplast was described (Shingles et al., 2001, 2002). This Fe^2+^ transport was inhibited by Zn^2+^, Cu^2+^, and Mn^2+^ in a competitive manner, and was activated by protons, similar to the reduction based iron (Fe^2+^) acquisition mechanism in roots. More recently, Fe-uptake experiments using bathophenantroline disulfonate (BPDS) on isolated sugar beet (*Beta vulgaris*) chloroplasts described that ferric (Fe[III]) citrate was preferred over ferrous (Fe[II]) citrate as an iron source (Solti et al., 2012). This Fe uptake was strongly connected to the photosynthetic performance of the chloroplast and subjected to negative feedback regulation. There are evidences of a reduction-based mechanism for chloroplast Fe-acquisition in strategy I and II plants, since the existence of a chloroplast ferric chelate oxidoreductase (FRO) has been demonstrated at the enzymatic activity level (Mikami et al., 2011; Solti et al., 2014). In intact barley (*Hordeum vulgare*) chloroplasts, FRO activity was induced by Fe deficiency under light, whereas it was repressed under dark conditions (Mikami et al., 2011). On isolated *B. vulgaris* chloroplast envelope fractions, Solti et al. (2014) showed that similar to the strategy I Fe-uptake in roots, a FRO enzyme, which clearly localized at the IE, and not the OE membrane, was responsible for complexed Fe(III) reduction and production of free Fe^2+^ for Fe uptake. This enzymatic activity was higher with NADPH than NADH, again suggesting the dependence of the Fe-acquisition mechanism on photosynthesis. The biphasic kinetics and its modification under Fe-deficient conditions also indicated the existence of high and low affinity mechanisms for Fe reduction. Since in the model plant *Arabidopsis thaliana* most likely only one member of the FRO family is localized in chloroplasts, this kinetics could be related to post translational modifications, differential splicing or to the existence of a yet unknown Fe-reduction mechanism.

**FIGURE 1:**
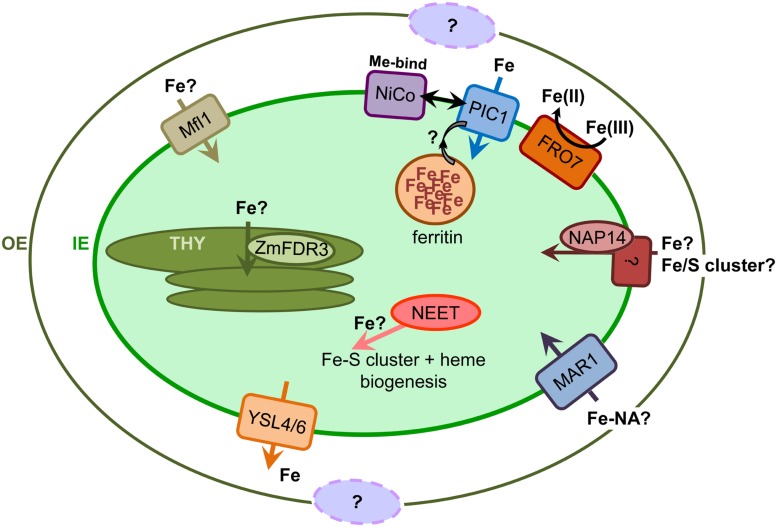
**Iron transport and homeostasis in chloroplasts.** Proteins and their possible functions in plastid Fe-transport and homeostasis are described throughout the text. Please note that for the sake of convenience, all transport proteins are depicted at the inner envelope membrane (IE), although only PIC1 could be localized unequivocally in the IE. Fe-transport proteins in the outer envelope (OE) are still unknown. For details on localization and structure of proteins compare **Table 1**. Me-bind, metal binding domain; THY, thylakoid membranes.

In *Arabidopsis*, the GFP-tagged FRO7 protein localizes to the chloroplast envelope (Jeong et al., 2008; **Figure 1**). Here, At-FRO7 is required for survival under Fe-limiting conditions, for efficient photosynthesis, and for proper chloroplast Fe acquisition in young seedlings (Jeong et al., 2008). The *fro7* loss-of-function mutants accumulate approximately 33% less iron per microgram of chlorophyll, show less Fe(III)-chelate reductase activity in isolated chloroplasts than the wild-type and have a chlorotic appearance when grown under Fe-limiting conditions. However, FRO7 is not Fe regulated (Mukherjee et al., 2006), the *fro7* loss-of-function mutants do not show visible growth phenotypes under standard conditions and their phenotype under Fe deficiency can be rescued by Fe addition, therefore suggesting the existence of redundant Fe uptake systems in chloroplasts and/or specialized plastids. In addition, staining of FRO7 promoter-GUS reporter lines revealed low expression in mature leaves (Jeong et al., 2008), supporting that its role may be ascribed to certain early developmental stages. The latter findings might also explain why - although containing several membrane-spanning domains (**Table 1**) - FRO7 is not found in envelope membranes within the AT_CHLORO database, which represents a comprehensive summary of proteomic analyses on chloroplast proteins with sub-plastidial annotation (Ferro et al., 2010).

**Table 1:** Proteins involved in chloroplast Fe acquisition and homeostasis as described throughout the text.

		Localization						
					
Name	AGI	ee	AT_C	cTP	α-TM	Putative function	Evidence	Reference
At-FRO7	At5g49740	ENV (GFP)	n.p.	y	10	Fe (III) reduction	*fro7* ko activity in yeast	Jeong et al., 2008
At-PIC1	At2g15290	IE (GFP, IMB)	IE	y	4	Fe uptake	*pic1* ko; *PIC1* ox yeast comp.	Duy et al., 2007b; Duy et al., 2011
Nt-PIC1	n.a.	ENV (GFP)	n.a.	y	4	Fe uptake	*Nt-PIC1* RNAi + ox yeast comp.	Gong et al., 2015
At-NiCo	At2g16800	ENV (GFP)	IE	y	6	Complex with PIC1 metal binding/transport	Interaction y2h metal binding domain	Duy et al., 2011; Eitinger et al., 2005
Os-ZN (=NiCo)	n.a.	THY?^1^ (GFP, ind. IMB)	n.a.	y	6–7	ROS protection	*Os-zn* pm	Li et al., 2010
At-YSL4	At5g41000	TON (GFP)	n.p.	n	14	Fe efflux	*ysl4 + ysl4ysl6* ko	Divol et al., 2013; Conte et al., 2013
At-YSL6	At3g27020	ENV (IMF, IMB) TON (GFP)	n.p.	n	14	Fe efflux	*ysl4 + ysl4ysl6* ko *YSL6* ox	Divol et al., 2013; Conte et al., 2013
At-MAR1/IREG3	At5g26820	ENV?^2^ (YFP)	n.p.	y	11	NA or Fe/NA uptake	*mar1* ko *MAR1* ox	Conte et al., 2009
At-NAP14	At5g14100	STR (GFP)	IE	y	0	Metal homeostasis and/or metal transport	*nap14* ko	Shimoni-Shor et al., 2010
At-Mlf1	At5g42130	–	IE	y	3–6	Fe transport	*mlf1* ko	Tarantino et al., 2011
Zm-FDR3	n.a.	C^3^ (ind. IMF)	n.a.	?	0	Possible transport of Fe-protein	*Zm-FDR3* ox in tobacco yeast comp.	Han et al., 2009
At-FER1	At5g01600	STR	STR^∗^	y	0	Protection against oxidative stress Fe storage	*fer1/3/4* triple ko	Ravet et al., 2009a;
At-FER2	At3g11050	STR^∗^	n.p.	y	0		*fer2* ko	Ravet et al., 2009b;
At-FER3	At3g56090	STR^∗^	STR	y	0		
At-FER4	At2g40300	STR^∗^/M (IMB)	STR	y	0	Metal homeostasis	*fer4* klao/frataxin mutant	Murgia and Vigani, 2015
At-NEET	At5g51720	C/M (GFP, IMB) STR (YFP, IMG)	STR	y	0	Fe–S/Fe cluster transfer	*neet* kd *in vitro* Fe-S/Fe transfer	Nechushtai et al., 2012; Su et al., 2013


*Names including plant species, *Arabidopsis* gene identifiers (AGI), the localization defined by experimental evidence (ee) and in proteomic studies (AT-CHLORO database, AT_C; Ferro et al., 2010), predicted chloroplast targeting peptides (cTP), and α-helical membrane-spanning domains (α-TM) according to the Aramemnon database (Schwacke et al., 2003), as well as putative functions and evidence described in the respective references are listed. ^1^: Please note that GFP-signals for Os-ZN have a ring-like appearance and immunoblots are only “indirect” (ind.), i.e., with antibodies directed against the GFP-tag. Thus, a localization of Os-ZN in chloroplast envelopes similar to its ortholog At-NiCo might be possible. ^2^: For At-MAR1/IREG3, YFP signals stain the entire chloroplast and cannot discriminate between thylakoid and envelope membranes. ^3^: The authors in Han et al., 2009 suggest presence of Zm-FDR3 in the thylakoid lumen, although “indirect” immunofluorescence against a MYC-tagged FDR3 protein only indicates general appearance in chloroplasts. ^∗^: for overview on chloroplast STR localisation of ferritin proteins in various plant species, see Briat et al., 2010a. At, *Arabidopsis thaliana*; C, chloroplast; comp., complementation; ENV, envelope; GFP, *in vivo* GFP targeting; IE, chloroplast inner envelope membrane; IMB, immunoblot; IMF, immunofluorescence; IMG, immunogold; kd, knock-down mutant; ko, knockout mutant; M, mitochondrium; n, no; n.a., not applicable; n.p., not present; Nt, *Nicotiana tabacum*; Os, *Oryza sativa*; ox, over-expressing line; pm, point mutation; ROS, reactive oxygene species; STR, chloroplast stroma; THY, thylakoid membranes; TON, tonoplast; y2h, yeast two hybrid; y, yes; Zm, *Zea mays*.*

In summary, these observations indicate that chloroplast Fe-transport across the outer envelope (OE) membrane occurs most likely via Fe(III) chelates, preferably Fe(III) citrate. Uptake across the IE, however, seems to occur mainly in the form of free Fe^2+^ and is accomplished by a reduction based and proton-driven mechanism similar to strategy I Fe-uptake in roots. However, other Fe-acquisition mechanisms cannot be excluded and also might depend on plant species, which prefer either strategy I or II mechanisms, different plastid types and/or developmental stages of tissues and organs. A certain diversification for Fe uptake has already been described in Gram-negative cyanobacteria - the evolutionary progenitors of chloroplasts - that import Fe(III) chelates or oxides across the outer membrane but at their plasma membrane either use a reduction based uptake of Fe^2+^ by the FeoB transporter or directly transport oxidized Fe^3+^ bound to periplasmic binding proteins *via* the Fut ABC transporter complex (see Kranzler et al., 2013, 2014).

### Chloroplast Iron Transport Proteins

Due to their endosymbiotic origin, chloroplasts as well as mitochondria are unique since they are surrounded by two membranes similar to their Gram-negative prokaryotic ancestors. Solute transporters in the IE membrane of chloroplasts were mainly derived from endomembranes of the eukaryotic host cell, and only few proteins have a prokaryotic origin, coming either from the membranes of the endosymbiont itself or from intracellular bacterial parasites (Tyra et al., 2007; Fischer, 2011). Surprisingly in contrast, the chloroplast OE largely originated from, and still resembles the outer membrane of the Gram-negative cyanobacterial-like endosymbiont (Block et al., 2007). In the chloroplast IE, numerous metabolite and ion transporter proteins have been identified and characterized thoroughly with respect to their physiological role and molecular mechanisms (Weber and Linka, 2011; Finazzi et al., 2015). These channels and transporters are hydrophobic, mainly α-helical membrane proteins facilitating the exchange of ions, and metabolic products with the cytoplasm. The characteristic channels of the outer membrane in Gram-negative bacteria and chloroplast OE, however, span the membrane in the form of β-strands that are organized to form a barrel-like pore structure (Duy et al., 2007a; Zeth and Thein, 2010).

Fe transport across the two envelope membranes of chloroplasts (import and export) is still not well understood (for overview see Nouet et al., 2011; Finazzi et al., 2015). However, research in the last decade has provided increasing evidence, which suggests that several families of proteins may play a role in Fe transport in chloroplasts. To date, proteins that shuttle Fe across the OE have not been identified, nevertheless, they might be similar to the ligand gated, TonB-dependent, β-barrel receptor channels in the outer membrane of Gram negative bacteria, i.e., FecA for Fe(III) citrate in *E. coli* or TBDTs (TonB-dependent transporters) in cyanobacteria (for overview see Chakraborty et al., 2007; Duy et al., 2007a; Kranzler et al., 2013). Direct and unequivocal experimental evidence for integration into the chloroplast IE membrane so far is only provided for the permease PIC1 (see below). However, several other Fe transporters, which have been localized to the chloroplast envelope, most likely are targeted to the IE membrane as well, while none of them has been ascribed to be integral to thylakoids. The current knowledge about Fe-transport and homeostasis proteins in chloroplasts is described below and summarized in **Figure 1** and **Table 1**.

#### PIC1 Participates in Chloroplast Fe Uptake and Plant Fe Homeostasis

The protein PIC1 (permease in chloroplasts 1) in *Arabidopsis* was identified in a screen for metal transport proteins as the first molecular component involved in plastid Fe-transport (**Figure 1**; Duy et al., 2007b). This integral membrane protein contains four membrane-spanning α-helices and was localized to the IE membrane of chloroplasts by *in vivo* GFP-targeting and immunoblot analysis (see **Table 1**). Interestingly PIC1 represents an ancient permease, clearly originating from the few proteins that were inherited by the cyanobacterial-like endosymbiont (see above; Duy et al., 2007b; Fischer, 2011). The function of PIC1 in Fe transport was verified by the growth complementation of a Fe uptake-deficient yeast strain where PIC1 could increase yeast cellular Fe-levels. Furthermore, PIC1 overexpression lines (PIC1ox) accumulate about 2.5-times more Fe in chloroplasts than wild-type plants (Duy et al., 2011). Thus, the functional analyses in yeast and the phenotypes of *pic1* loss-of-function mutants and PIC1ox lines clearly show that PIC1 participates in plastid Fe-uptake as well as Fe-homeostasis pathways throughout the plant. Plants lacking PIC1 are characterized by a strong dwarf, albino phenotype resembling Fe-deficiency symptoms in plants. In addition, *pic1* mutant plants display disrupted leaf mesophyll organization and a severely impaired development of chloroplasts, which suggests Fe shortage within the organelle (Duy et al., 2007b). Only recently, the function of PIC1 in plastid Fe-transport has been further supported by studies of *PIC1* knockdown and overexpression lines in tobacco (*Nicotiana tabacum*) plants, which showed similar phenotypes to the *Arabidopsis* PIC1 mutants (Gong et al., 2015). Furthermore, Nt-PIC1 was also able to complement growth of Fe-deficient yeast.

Surprisingly, on the one hand *Arabidopsis pic1* knockout plastids/chloroplasts in meristems and leaves show an accumulation of ferritin protein clusters, which most likely are loaded with Fe (Duy et al., 2007b). In addition to their role as Fe-storage proteins during germination, plastid-intrinsic ferritins function in Fe sequestration when Fe is present in excess, thereby protecting cells against oxidative damage (Ravet et al., 2009a; Briat et al., 2010b). On the other hand, the phenotype of PIC1ox mutants clearly resembles that of ferritin loss-of-function plants under Fe excess, showing severe defects in flower and seed development that result in reduced seed yield and germination rates (Ravet et al., 2009a; Duy et al., 2011). Flowers of PIC1ox mutants contain more Fe, while other transition metals are unaffected, whereas seeds show a significant reduction in the concentration of this metal. Furthermore, PIC1 transcript levels are slightly up-regulated in leaf ferritin knockouts (Ravet et al., 2009a). All these findings detailed above point toward a close reciprocal correlation between PIC1 and ferritin which might be explained as follows: (i) In leaves of *pic1* loss-of-function plants, chloroplast-intrinsic Fe levels are low and therefore thylakoid membrane systems are degraded. However, at least in seed and meristem tissues, a plastid Fe-uptake bypass pathway exists, which allows germination and slow growth of *pic1* lines. When leaves mature, Fe-uptake mediated by PIC1 becomes dominant, and cytosolic Fe-levels increase transiently due to the blockage in chloroplast Fe-uptake of *pic1* plants, which in turn leads to oxidative stress and induction of ferritin expression. The plastid-intrinsic ferritin accumulating in *pic1* knockouts thus is most likely induced by a cytosolic signal transduction pathway and stores Fe coming from degraded thylakoids. (ii) In contrast, plastid levels of free Fe-ions significantly increase in both, PIC1ox lines under Fe sufficient and ferritin knockouts under Fe-excess growth conditions. In this situation, plants have to cope with plastid-intrinsic oxidative stress that leads to similar phenotypes concerning flower and, in particular, seed development. Transcriptional profiling of *pic1* and PIC1ox plants revealed that major changes occurred in genes related to metal transport and homeostasis and future studies might contribute to unravel plastid- and nucleus-driven signaling processes under disturbed Fe homeostasis (Vigani et al., 2013).

The permease PIC1 has also been described as Tic21 (translocon at the IE membrane of chloroplasts of 21 kDa), a putative IE translocon component, which could participate in import of nuclear-encoded plastid proteins from the cytosol (Teng et al., 2006). The *Arabidopsis* protein Tic21, which is identical to PIC1, was identified by forward genetics using a screen for mutants defective in chloroplast protein import (Sun et al., 2001). The proposed function of Tic21/PIC1 as essential translocon component in the chloroplast IE by Teng et al. (2006) is mainly based on the accumulation of unprocessed plastid precursor proteins in *tic21/pic1* knockout lines, defects in IE protein translocation in isolated chloroplasts of a sub-lethal *tic21/pic1* mutant, and co-immunoprecipitation of Tic21/PIC1 with other major protein import translocon components. However, a direct functional analysis of Tic21/PIC1 for protein transport is lacking, and analyses of Duy et al. (2007b) failed to detect residual plastid precursor proteins in identical *pic1/tic21* mutant lines. Moreover, plastid-localized ferritin was not only accumulating in *pic1/tic21* plastids, but also properly processed, indicating that PIC1/Tic21 is not involved in protein translocation (for detailed discussion see Duy et al., 2007b). In 2009, a protein complex of about 1 MDa was identified at the chloroplast IE membrane containing the putative translocon channel Tic20, a large fraction (about 900 kDa) of yet unidentified membrane proteins, and also small amounts of Tic21/PIC1 (Kikuchi et al., 2009). The authors hypothesized that this complex might function as a general TIC protein translocation core complex, where Tic21/PIC1 is loosely associated to the periphery. More recent publications, however, which lead to the identification of the other proteins in this putative TIC protein translocation core, demonstrated that Tic21/PIC1 does not co-purify with this 1 MDa complex (Kikuchi et al., 2013; Nakai, 2015). Therefore, the previously described attachment of PIC1/Tic21 to TIC translocon component proteins such as Tic20 might have been generated by the high membrane protein density in the chloroplast IE, rather than by specific protein interactions.

PIC1/Tic21 displays a close phylogenetic relationship to cyanobacterial proteins annotated as putative permease components functioning in solute and/or ion transport across membranes (Duy et al., 2007b). The ortholog of PIC1/Tic21 in *Synechocystis* sp. PCC 6803, Sll1656, can functionally complement *pic1*/*tic21* loss-of-function plants (Lv et al., 2009) and the growth of Fe uptake-deficient yeast mutants (Duy et al., 2007b). In contrast, PIC1/Tic21 shares little sequence similarity to protein translocon channels in the chloroplast IE (e.g., Tic20) or to those in the inner membrane of mitochondria (Tim17 and Tim23; Inaba and Schnell, 2008; Balsera et al., 2009), although all of them share a similar structure containing four predicted α-helical membrane domains. According to Gross and Bhattacharya (2009), however, a possible dual function of PIC1/Tic21 in iron transport and protein import is not mutually exclusive, arguing that the beneficial effect of new functional properties (such as protein import) would be an evolutionary addition to its function as an ancient solute permease of cyanobacterial origin.

In addition to the PIC1 function in Fe accumulation in yeast cells (Duy et al., 2007b; Gong et al., 2015), and in *Arabidopsis* chloroplasts when over-expressed (Duy et al., 2011), the interaction of PIC1 with the putative metal transport protein NiCo (Eitinger et al., 2005) at the plastid IE membrane, points to a role in Fe transport (Duy et al., 2011). Furthermore, transcript levels of At2g16800 - one of the two *Arabidopsis* NiCo genes - were increased in *PIC1*ox mutant flowers (Duy et al., 2011). *In vivo* GFP-targeting of the corresponding protein shows characteristic ring-shaped signals of chloroplast envelope proteins, which is supported by the occurrence of At2g16800 in IE proteomics (see **Table 1**; Eitinger et al., 2005; Ferro et al., 2010; Duy et al., 2011). A point mutation of the *NiCo* ortholog in rice (named Os-ZN, for zebra-necrosis protein) resulted in a protein that - although most likely mislocalized to thylakoids (see above and **Table 1**) - caused a yellow-stripe, necrotic leaf phenotype (Li et al., 2010), which was light-dependent and related to ROS production. Therefore, a role of PIC1-NiCo in an iron translocon complex at the IE of plastids is very likely (see **Figure 1**; Duy et al., 2011). Given that NiCo proteins contain specific metal binding domains (Eitinger et al., 2005), a possible molecular mechanism for PIC1-NiCo interaction in *Arabidopsis* IE membranes, could involve Fe binding by At-NiCo and its subsequent transfer to the permease PIC1. However, more direct functional assays for metal transport still need to be established to study the molecular mechanisms in detail.

#### Other Proteins Participating in Fe Transport Across the Chloroplast Envelope

Two transporters from the “yellow stripe 1-like” family of *Arabidopsis*, YSL4 and YSL6, have been characterized as potential plastid Fe-efflux transporters (**Figure 1**; Divol et al., 2013). Both genes are up-regulated in response to Fe excess and at least one of them, YSL6, was unequivocally localized to the chloroplast envelope by immunoblot and immuno-fluorescence analysis (**Table 1**; Divol et al., 2013). However, if YSL6 integrates into the OE or IE, still remains an open question, since the protein could not be identified in proteomic analyses of chloroplast proteins. Furthermore, neither YSL4 nor YSL6 contain a classical, N-terminal chloroplast targeting peptide (see **Table 1**), a feature which is more common for OE than for IE proteins. Phenotypical characterization of single and double knockout mutants showed that Fe accumulated in the chloroplasts, and this occurred concomitantly with an increase in ferritin, whereas ubiquitous over-expression of YSL4 and YSL6 caused sensitivity to Fe and a decrease of Fe in chloroplasts (Divol et al., 2013). In addition, the coordinated expression of these YSL genes with ferritin genes in embryos and senescent leaves prompted the authors to propose their physiological role in detoxifying Fe during plastid differentiation in embryogenesis and senescence (Divol et al., 2013). The role of these transporters, however, remains controversial since due to proteomic data and GFP-targeting they have also been associated to transport of metal ions across tonoplast and ER membranes (see **Table 1**; Conte et al., 2013).

The *Arabidopsis* “multiple antibiotic resistance 1/iron regulated 3” (MAR1/IREG3) protein, which most likely transports aminoglycoside antibiotics into plastids and has been localized to chloroplasts via targeting of a YFP-tag (**Table 1**), also plays a role in cellular Fe homeostasis (Conte et al., 2009; Conte and Lloyd, 2010). Because MAR1/IREG3 expression is down-regulated by Fe deficiency and MAR1 overexpressing plants show leaf chlorosis that can be rescued by Fe addition, it has been proposed that MAR1/IREG3 may play a role in Fe chelation, storage, or sequestration (Conte et al., 2009). In addition, MAR1/IREG3 belongs to the IREG/ferroportin transporter family that includes IREG1/FPN1 and IREG2/FPN2, which mediate metal transport across the plasma membrane in the root stele and tonoplast, respectively (Schaaf et al., 2006; Morrissey et al., 2009). Interestingly, aminoglycosides can use polyamine inward transport systems to enter eukaryotic cells (Van Bambeke et al., 2000), and one of the most important natural chelators of Fe, nicotianamine (NA) is a polyamine (for overview, see Curie et al., 2009). These observations have prompted the hypothesis that MAR1/IREG3 may transport NA or an Fe-NA complex into the plastid (**Figure 1**; Conte et al., 2009; Conte and Lloyd, 2010). Further indications of the role of this protein in Fe homeostasis were found in a study of quantitative trait locus (QTL) mapping for *Arabidopsis* seed mineral concentrations, where this gene was found in two QTLs associated with the seed Fe trait (Waters and Grusak, 2008).

A bioinformatics approach using cyanobacterial Fe-transporter genes as queries revealed the existence of a plastid-localized, non-intrinsic ABC transporter protein, NAP14 in *Arabidopsis*. *At-NAP14* (also named ABCI11 according to ABC transporter nomenclature [Verrier et al., 2008]) encodes for a non-membrane intrisic, nucleotide binding domain (NBD) subunit of an ABC transporter complex, similar to the FutC unit of the FutABC Fe transporter in cyanobacteria (Shimoni-Shor et al., 2010). Although *in vivo* GFP targeting by Shimoni-Shor et al. (2010) shows signals in the chloroplast stroma, the At_CHLORO database (Ferro et al., 2010) associates this protein to the IE membrane, indicating an attachment of NAP14 to a membrane-intrinsic protein component (see **Figure 1**, **Table 1**). The iron concentration in shoots of *nap14* loss of function mutants is dramatically increased (18 times higher than in wild-type plants), whereas in roots it is approximately 50% lower. Moreover, this mutant showed damage to chloroplast structures, exhibited severe growth defects, strong chlorosis and a deregulation of the Fe-homeostasis genes (Shimoni-Shor et al., 2010). Based on these findings, either a function in regulating plastid Fe-homeostasis or a direct role as part of a plastid Fe ABC-transporter complex have been proposed. NAP14 could also be involved in Fe–S cluster biogenesis similar to NAP7/SufC, a stroma localized NBD-NAP protein (Xu and Möller, 2004; Balk and Schaedler, 2014). Furthermore, high-throughput proteomic studies have demonstrated the presence of several ABC transporters of still unknown function in the IE membrane, including some “half-size” proteins such as NAP8/ABCB28 (consisting of 1 membrane-intrinsic permease and 1 soluble NBD domain [Verrier et al., 2008]), as well as NAP13/ABCI10, a relative to NAP14 (Ferro et al., 2010; Gutierrez-Carbonell et al., 2014), whose roles in plastid Fe homeostasis deserve further studies. These proteomic studies also indicated the presence of the half-size ABC transporter STA1/ATM3/ABCB25 in the chloroplast envelope. Initially, this transporter was associated to Fe–S cluster export from the mitochondria (see Bernard et al., 2013) but recently, its role in glutathione polysulfide export from the mitochondria for Fe–S cluster assembly in the cytosol has been described (Schaedler et al., 2014). Although STA1/ATM3/ABCB25 is clearly localized in the inner mitochondrial membrane, its presence in chloroplast envelope proteomes suggests a possible dual localization that may be verified by future studies.

Another reasonable candidate for Fe import into plastids is At-Mfl1 (mitoferritin-like1). This gene was found in an attempt to uncover mitochondrial Fe-transporters in *Arabidopsis* using the template mitoferrin 2 (MFRN2), a mitochondrial Fe importer in non-erythroid cells from *Danio rerio* (zebra fish; Tarantino et al., 2011). At-Mfl1 belongs to the mitochondrial carrier family, is annotated as an IE protein in the AT_CHLORO database, has been detected in the envelope proteome of various plant species [see Ferro et al., 2010, Supplemental Table S10 and references therein], and contains a predicted chloroplast targeting peptide (**Table 1**). The expression of this gene is mainly localized in rosette leaves, it is upregulated with Fe excess and correlates with that of genes coding for proteins involved in chloroplast metabolism including PIC1 (Tarantino et al., 2011). *At-Mlf1* loss-of-function mutantlines present lower seedling and leaf Fe-concentrations than wild-type plants and a reduced expression of *At-ferritin1* (Tarantino et al., 2011), suggesting a putative role for Mfl1 as a chloroplast Fe-transporter (**Figure 1**). The observation that mutants with a loss-of-function are viable and fertile implies that the role of At-Mfl1 is redundant or that it plays a specific function only when Fe is in excess.

In maize, differential display screening allowed the identification of the Fe-deficiency inducible gene Zm-FDR3 (*Zea mays* Fe-deficiency related 3; Yin et al., 2000). Yeast complementation studies indicated that this protein can transport Fe and/or Cu (Han et al., 2009). Because orthologs of this gene were not found in any strategy I plant including *Arabidopsis*, tobacco was used to study Zm-FDR3 function. In transgenic tobacco, overexpressing a MYC-tagged Zm-FDR3, this protein was presumably localized to chloroplasts by immunofluorescence labeling directed against the tag (**Table 1**). Since the protein contains no predicted membrane-spanning domains, it could be most likely in the stroma or the thylakoid lumen. Based on an *in silico* prediction, the latter is proposed by Han et al. (2009) (**Figure 1**). Transgenic tobacco plants, expressing Zm-FDR3 displayed higher seed Fe concentration, lower chlorophyll concentration but higher photosynthetic performance, therefore suggesting a role in plastid Fe-homeostasis. This protein does not have homology to other proteins in higher plants but contains domains similar to members of the bacterial type III secretion system, involved in the secretion of effector proteins into a host cell, which might imply the involvement of Zm-FDR3 in the transport of an Fe-containing protein (Han et al., 2009). The existence of such a mechanism in plants and specifically in thylakoids is untypical but plausible. For instance, the assembled Rieske Fe–S protein of the cytochrome b6/f complex can utilize the TAT protein import pathway for its translocation into the thylakoid membrane (Molik et al., 2001). However, knowledge on Zm-FDR3 structure and exact localization is very limited, since the protein contains no predicted membrane-spanning domains and no chloroplast targeting peptide (see **Table 1**).

## Plastid Fe Homeostasis

As a sink for most of the cellular Fe, plastids are likely to be involved in sensing and regulation of Fe concentration within the whole plant and changes in the plastid Fe-status may trigger different adaptation responses depending on the plant developmental stage (Vigani et al., 2013). In spite of this important putative role, little is known about processes governing Fe homeostasis and signaling in plastid organelles and in their communication with other cellular compartments. For general and tissue-specific subcellular Fe distribution see (Kim et al., 2006; Roschzttardtz et al., 2013; Mary et al., 2015).

### Fe Trafficking and Storage in Plastids

Ferritins are conserved eukaryotic proteins and their homo-oligomeric structure of 24 assembled subunits forms a hollow core that can accommodate up to 4,000 Fe(III) ions (for overview see Briat et al., 2010a,b). Plant ferritins are nuclear encoded and *Arabidopsis* contains four ferritin genes (**Table 1**), three of which encode for chloroplast targeted proteins, and one for a protein targeted to seed plastids (Petit et al., 2001; Ravet et al., 2009b). For an overview on plastid stroma localization of ferritin proteins (**Figure 1**) in various plant species we refer to Briat et al. (2010a). Furthermore ferritins - in particular At-FER4 via immunoblots (see **Table 1**) - have also been described in mitochondria (Tarantino et al., 2010; Murgia and Vigani, 2015). In contrast to seed tissue, in mature leaf chloroplasts ferritin proteins are less abundant, however, they accumulate in chloroplasts under Fe excess conditions (Briat et al., 2010b) and thereby play an important role in buffering Fe in its free ionic form and preventing oxidative stress (Ravet et al., 2009a). Whereas in humans the chaperone involved in Fe delivery to ferritin has been discovered (Shi et al., 2008), in plants the machinery involved in the delivery of Fe to ferritin is largely unknown (Briat et al., 2010a,b). Given the active redox nature of Fe, the involvement of metallo-chaperones or chelating molecules in this process is more than likely, and might be similar to the CCS chaperone that delivers Cu to the Cu/Zn superoxide dismutase (SOD) in plastids (Cohu et al., 2009). The mechanism underlying the release of Fe from ferritin is even more enigmatic. On one hand, *in vitro* studies with animal ferritins suggest that this release requires Fe chelators or reducing agents. On the other hand, *in vivo* studies have demonstrated Fe release by proteolytic degradation of the protein (see Briat et al., 2010a,b for details). Regarding the observed correlation of PIC1 and ferritin function in chloroplasts (see above), a role for PIC1 in handover of imported Fe to the ferritin protein shell might be possible as well.

Plastid organelles harbor an independent pathway for the assembly of Fe–S clusters, the SUF (sulfur mobilization) system, which derives from their cyanobacterial ancestor (Balk and Pilon, 2011). Several components of this pathway have been elucidated and include a cysteine desulfurase (NFS2), scaffold proteins including the SUFBCD complex and NFU proteins, and the cluster transfer and insertion proteins (SUFA, HCF101, GRXS14 and GRXS16; see Couturier et al., 2013; Balk and Schaedler, 2014 for overview). However, the proteinaceous source of Fe for plastid Fe–S cluster synthesis is still unknown. Another Fe–S cluster protein that has been described to play a role in Fe homeostasis in the chloroplast belongs to the NEET family, which is involved in a large array of biological processes in mammalian cells. Although their mode of function is largely unknown, their 2Fe–2S cluster is labile, thereby allowing a role in Fe–S/Fe transfer within cells (Zuris et al., 2011). The *Arabidopsis* protein, At-NEET, most likely is involved in Fe–S/Fe cluster transfer to an acceptor protein and initially was dually localized to chloroplasts and mitochondria by GFP-targeting and immunoblot analysis (**Table 1**; Nechushtai et al., 2012). This protein has been categorized as a plastid protein in AT_CHLORO, and its sub-organellar localization within the chloroplast stroma (immunogold labeling) has been recently reported (Su et al., 2013). At-NEET knockdown plants obtained by RNA interference (RNAi) accumulate more Fe and present higher ROS levels (Nechushtai et al., 2012). The growth of At-NEET knockdown seedlings is insensitive to high, but sensitive to low Fe-levels, strongly suggesting that this protein is involved in Fe transfer, distribution, and/or management. Furthermore, At-NEET knockdown mutants present a dramatic decrease in catalase abundance, a heme enzyme that detoxifies ROS, suggesting also a role of this protein in the supply of heme Fe (Nechushtai et al., 2012).

Another candidate to maintain Fe homeostasis in the plastid is NA. This polyamine has a role in metal chelating in phloem sap, vacuoles and cytoplasm (Pich et al., 1997; Haydon et al., 2012). The presence of NA in plastids has not been studied so far. However, as pointed out by Divol et al. (2013), the leaves from the tomato mutant *chloronerva*, which does not contain NA, present electron dense inclusions of Fe and phosphor in the chloroplast that are absent in wild-type and do not correspond to ferritin (Becker et al., 1995). Thus, these inclusions might indicate a role of NA in maintaining Fe in a soluble form in plastids.

### Cross Talk with Vacuoles and Mitochondria

Both, chloroplasts and mitochondria are rich in Fe-containing proteins and although they are most likely major control points in the Fe homeostasis network, little is known about the mechanisms involved in their communication (see Vigani et al., 2013). In these two compartments, Fe is a major constituent of proteins belonging to their respective electron transfer chains and therefore Fe deficiency has a strong impact on their performances. Under Fe deficiency, the mitochondrial electron transport chain undergoes modifications aiming to bypass the Fe-rich complexes, mainly complex I, by the action of alternative NAD(P)H dehydrogenases (Vigani et al., 2009; Vigani and Zocchi, 2010) and this occurs concomitantly with increases in the glycolytic pathway (López-Millán et al., 2000; Zocchi et al., 2007). At the same time, Fe deficiency has a strong impact on thylakoid PSI, the highest Fe sink of the photosynthetic electron chain, and causes a remodeling of the antenna complexes that alters the stoichiometry of both PSI and PSII (Abadia, 1992; Moseley et al., 2002). While in the green microalgae *Chlamydomonas reinhardtii*, mitochondria are more resistant than chloroplasts to Fe deficiency, therefore suggesting a preference for Fe delivery to mitochondria under Fe starvation (Naumann et al., 2007; Nouet et al., 2011), nothing is known about the preference for Fe allocation between these compartments in vascular plants. Indirect evidences exist to support the existence of a cross talk between mitochondria and chloroplasts since for instance loss of function mutants of MIT, the mitochondrial iron transporter for Fe uptake, show lower chlorophyll content and altered ferritin gene expression (Bashir et al., 2011).

The phenotype of the double knockout mutant *ysl4ysl6* suggests a close communication between vacuoles and plastids in embryo tissue during seed germination. In this mutant, the expression of the transporters NRAMP3 and NRAMP4, which are responsible for vacuolar Fe-export during germination, is dramatically down-regulated (Divol et al., 2013). In addition, the double *nramp3 nramp4* mutant does not contain FER2, the seed stable ferritin isoform, which suggests a decrease of Fe in the plastid (Ravet et al., 2009b), and this has been explained by the prevention of Fe from exiting the vacuole (Divol et al., 2013). However, as previously discussed in the same publication, the expression of these genes may not occur in the same cells and therefore the cross-talk between the vacuolar NRAMP3/4 and the plastid YSL4/6 must involve sensing of Fe in the plastid and signaling to adjust Fe release from the vacuole.

### The Role of Plastids in Seed Fe-Filling

Metal loading in seeds depends on both, root soil uptake and remobilization from source tissues, including senescent leaves. Senescent organs are a major source for nitrogen but little is known about their contribution to metal remobilization. As the sink for most of the plant Fe, it is tempting to speculate that plastids may play a role by providing Fe in this process. During leaf senescence, the chloroplast is one of the first organelles to be degraded. This occurs gradually: initially pigment degradation occurs in the chloroplast whereas the degradation of stromal proteins takes place in the vacuole. These processes reduce the number and size of plastids and allow for further degradation via chlorophagy (Wada and Ishida, 2009; Pottier et al., 2014). RuBisCo from the chloroplast stroma of senescing leaf cells is a major source of remobilized nitrogen for seed filling. In this context the role of Fe-rich plastid proteins in Fe remobilizing should be further explored. Furthermore, chloroplasts could also provide Fe when this metal is limited in the environment because autophagy for nutrient remobilization can be triggered upon nutrient starvation and stress (Pottier et al., 2014). These hypotheses are consistent with the strong expression of YSL6 in senescent leaves and its putative role in controlling Fe release from the plastid during senescence (Divol et al., 2013). On the other hand, transcriptomic analyses have also shown that senescence occurs concomitantly with an upregulation of vacuolar Fe-transporters such as NRAMP3 (Thomine et al., 2003; Pottier et al., 2014), suggesting that in physiological conditions both the vacuole and the plastid can take part in Fe remobilization.

## Concluding Remarks

In summary, research in the last decade has led to the discovery of several Fe-transport proteins in plastids. Detailed phenotypic analyses of the corresponding mutants in the model plant *Arabidopsis*, already allow us to obtain a quite accurate description of the role of these proteins in plant Fe homeostasis and general physiology. Unfortunately, exact and unequivocal localization at the sub-cellular and sub-organelle level is still missing for most of these membrane proteins. Future research thus definitely requires unbiased and independent approaches such as *in vivo* GFP targeting in combination with specific immuno-localization and membrane proteomic analyses as well as *in silico* predictions of targeting signals and *in vitro* import assays into organelles. Furthermore, Fe-transport proteins in the chloroplast OE still await their discovery. Major questions to be answered include the exact transport mechanisms, their respective structure/function relationships and the nature of their substrates such as free Fe–ions or chelates. Established - e.g., yeast, *E. coli* -, but also new heterologous systems for functional transport assays such as Gram-positive *Lactococcus lactis*, might lead to more detailed answers in future research.

## Author Contributions

AFL-M, DD, and KP wrote the manuscript.

## Conflict of Interest Statement

The authors declare that the research was conducted in the absence of any commercial or financial relationships that could be construed as a potential conflict of interest.
